# Spatial effects of township health centers’ health resource allocation efficiency in China

**DOI:** 10.3389/fpubh.2024.1420867

**Published:** 2024-08-16

**Authors:** Nannan Meng, Keyi Shen, Xinyue Zheng, Chengcheng Li, Xinhao Lin, Tong Pei, Dan Wu, Xuehui Meng

**Affiliations:** School of Humanities and Management, Zhejiang Chinese Medical University, Hangzhou, Zhejiang, China

**Keywords:** township health centers (THCs), resource allocation, data envelopment analysis (DEA), spatial auto-correlation, Spatial Dubin Model

## Abstract

**Introduction:**

China is a large agricultural nation with the majority of the population residing in rural areas. The allocation of health resources in rural areas significantly affects the basic rights to life and health for rural residents. Despite the progress made by the Chinese government in improving rural healthcare, there is still room for improvement. This study aims to assess the spatial spillover effects of rural health resource allocation efficiency in China, particularly focusing on township health centers (THCs), and examine the factors influencing this efficiency to provide recommendations to optimize the allocation of health resources in rural China.

**Methods:**

This study analyzed health resource allocation efficiency in Chinese rural areas from 2012 to 2021 by using the super-efficiency SBM model and the global Malmquist model. Additionally, the spatial auto-correlation of THC health resource allocation efficiency was verified through Moran test, and three spatial econometric models were constructed to further analyze the factors influencing efficiency.

**Results:**

The key findings are: firstly, the average efficiency of health resource allocation in THCs was 0.676, suggesting a generally inefficient allocation of health resources over the decade. Secondly, the average Malmquist productivity index of THCs was 0.968, indicating a downward trend in efficiency with both non-scale and non-technical efficient features. Thirdly, Moran’s Index analysis revealed that efficiency has a significant spatial auto-correlation and most provinces’ values are located in the spatial agglomeration quadrant. Fourthly, the SDM model identified several factors that impact THC health resource allocation efficiency to varying degrees, including the efficiency of total health resource allocation, population density, PGDP, urban unemployment rate, *per capita* disposable income, *per capita* healthcare expenditure ratio, public health budget, and passenger traffic volume.

**Discussion:**

To enhance the efficiency of THC healthcare resource allocation in China, the government should not only manage the investment of health resources to align with the actual demand for health services but also make use of the spatial spillover effect of efficiency. This involves focusing on factors such as total healthcare resource allocation efficiency, population density, etc. to effectively enhance the efficiency of health resource allocation and ensure the health of rural residents.

## 1 Introduction

Being healthy is a fundamental right of individuals, and everyone possesses the opportunity and capability to attain “health equity.” The allocation of health resources in rural regions significantly impacts the preservation of the most essential rights to life and health for rural inhabitants. To uphold health equity and societal stability, an increasing number of nations are prioritizing the advancement of rural healthcare services, and research on medical and healthcare in rural areas is becoming progressively comprehensive. Scholars such as Almeida have investigated the health services accessibility of rural residents through their research on 27 remote rural municipalities in Brazil (1). Additionally, Bautista Gómez and others have examined the case of Sumapas in the rural area of Bogotá, Colombia, to explore how to ensure fair access to high-quality healthcare services for rural populations (2). Meanwhile, Carey and colleagues have advocated in their research on “which primary healthcare services should be accessible to residents in rural and remote areas of Australia” that everyone should have access to primary healthcare core services at all times (3).

China has consistently prioritized rural healthcare and actively advanced rural health initiatives, while also implementing numerous practical and viable policies for public health (4, 5). From 2009 to 2011, the Chinese government invested 4.448 billion yuan for rural sanitation improvement through a 3-year health reform program (6).These measures and investments not only have the potential to enhance the health status of rural inhabitants (7) but also to stimulate economic and social progress in rural regions (8). They can also contribute to bridging the urban–rural healthcare gap (9), thereby fostering the holistic development of the healthcare sector (10). Nevertheless, many scholars perceived significant room for improvement in China’s rural health development. For instance, Hu et al. argued in their study that the reimbursement policies of the New Cooperative Medical Scheme may restrict rural residents’ access to healthcare services (11). Additionally, scholars like Liu have identified the substantial challenge that China faces in maintaining equity and efficiency in healthcare resource allocation and utilization amidst rapid economic growth in rural areas (12).

The township health centers (THCs) occupy a central position within China’s rural three-tier medical and health service network. Serving as the crucial link between county-level medical institutions and village clinics, they play a vital role in safeguarding the health of rural residents (13). The primary responsibility of THCs is to deliver fundamental medical services, encompassing disease prevention, diagnosis, treatment, and rehabilitation (14). Alongside undertaking public health services and management functions, including planned immunization, health education, maternal and child health care, and health supervision (15). Consequently, China is actively advancing the THC construction project and continually enhancing the three-tier medical and health service system at the county, township, and village levels to safeguard the health of rural populations. Despite the government’s escalating investment in THCs’ basic resources following the healthcare reform, the utilization of THC medical services within the rural medical and health service system has progressively dwindled (16), with its medical service output also proved suboptimal (17). For instance, Chen et al. (18) found in their research that the efficiency of THC health resource allocation in China is low, and there are significant regional differences in health resource allocation. Xu et al. (19) asserted that there is a problem with output speed being lower than input speed in THCs in China. Furthermore, Jiang et al. (20) discovered that although the medical community can significantly increase the scale efficiency of THC expansion, they cannot effectively improve THC technical efficiency and pure technical efficiency. Moreover, numerous researchers have identified developmental challenges faced by THCs in China, including insufficient number of health personnel, shrinking service content, decreased patient satisfaction and resident trust (21, 22).

China has made remarkable progress in the development and research of rural health initiatives. For example, numerous studies have highlighted significant advancements in the construction of the rural health service system, including the establishment and coverage of county-level medical institutions, township health centers, and village clinics, for these facilities did have improved accessibility to basic medical services for rural residents (23, 24). Furthermore, many scholars have explored the training, recruitment, and incentive mechanisms to attract and retain excellent health talents to work in rural areas (25). Alongside these qualitative researches, there have been various quantitative analyses of rural health in China, contributing to a comprehensive understanding of the subject. For example, scholars such as Ao have conducted research on the equity of resource allocation in China’s rural three-tier healthcare system through the Gini coefficient, health resource density index, and Theil index (26). Li et al. (27) have analyzed the effects of tiered healthcare services delivery on cost control and quality improvement in rural China using interrupted time series data. Qiu et al. (28) have explored the impact of China’s healthcare system reform on improving healthcare services by evaluating the urban–rural gap in the medical system in Dalian from 2008 to 2017.

However, when examining quantitative research on the health status of rural areas in China both domestically and internationally, it becomes apparent that there is a lack of specialized research and analysis on the efficiency of health resource allocation in rural areas. Most efficiency studies conducted in rural areas are either based on cross-sectional data or are confined to the provincial level (29–31). The few efficiency studies at the national level only briefly mention the efficiency of health resource allocation without further analysis of it, such as the spatial agglomeration of efficiency and factors affecting it (12, 32).

To effectively demonstrate the efficiency of THC health resource allocation at the national level in China and conduct a more specific analysis of rural health resource allocation in China, this study fully utilized calculation software such as Microsoft Office Excel, MathWorks Matlab R2021b, and StataCorp LLC Stata16.0, and combined these with the super-efficiency SBM model and global Malmquist model to calculate the THCs’ health resource allocation efficiency of 29 provinces (autonomous regions, municipalities) in China from 2012 to 2021. In addition, this study used Moran’s Index to test the spatial auto-correlation and constructed spatial econometric models based on the adjacency matrix to analyze the factors that affect the efficiency of THC health resource allocation in various provinces, thereby proposing scientific references for improving the efficiency of rural health resource allocation in China.

The innovations of this research lie in: firstly, by utilizing THCs’ health resource data that can effectively represent the medical and health status in rural areas, this study achieved a national-level study on the efficiency of rural health resource allocation. Secondly, this study utilized panel data of THCs in various provinces from 2012 to 2021 to study the dynamic changes in the efficiency of THC health resource allocation. Finally, this study creatively combined spatial econometric models in the analysis of the efficiency of health resource allocation, thereby enriching the research methods of health resource allocation.

## 2 Materials and methods

### 2.1 Data source and variable selection

The spatial basic unit for this study is the provincial-level administrative regions in China from 2012 to 2021. The study chose 29 provinces, autonomous regions, and municipalities directly under the central government in China, out of the 34 provincial-level administrative regions (Due to the lack of relevant research data from Beijing, Shanghai, Hong Kong, Macao, and Taiwan, these 5 regions were excluded in our research). The specific names and numerical labels of each research region are provided in Table 1.

**Table 1:** Efficiency of THC health resource allocation in each province.

DMU	Regions	2012	2013	2014	2015	2016	2017	2018	2019	2020	2021	Mean
1	Tianjin	1.027	1.031	0.922	1.022	1.014	1.005	0.952	1.030	1.004	1.008	1.002
2	Hebei	1.010	0.936	0.804	0.779	0.886	0.588	0.548	0.463	0.365	0.286	0.667
3	Shanxi	0.363	0.345	0.320	0.305	0.302	0.331	0.305	0.268	0.196	0.130	0.287
4	Inner Mongolia	0.332	0.350	0.325	0.299	0.324	0.332	0.297	0.212	0.179	0.156	0.281
5	Liaoning	0.473	0.465	0.440	0.418	0.426	0.464	0.430	0.357	0.244	0.208	0.392
6	Jilin	0.308	0.233	0.220	0.198	0.203	0.241	0.255	0.186	0.119	0.121	0.208
7	Heilongjiang	0.489	0.531	0.463	0.486	0.514	0.520	0.375	0.329	0.216	0.197	0.412
8	Jiangsu	0.717	0.815	0.843	0.870	0.851	1.030	0.928	1.011	0.748	0.757	0.857
9	Zhejiang	1.016	0.941	1.011	1.013	0.932	1.010	1.011	1.031	0.664	0.708	0.934
10	Anhui	0.704	0.723	0.717	0.689	0.663	0.720	0.689	0.679	0.529	0.439	0.655
11	Fujian	0.714	0.665	0.600	0.565	0.546	0.520	0.516	0.524	0.385	0.375	0.541
12	Jiangxi	1.013	0.840	0.664	0.673	0.665	0.699	0.664	0.613	0.525	0.526	0.688
13	Shandong	1.004	0.630	0.624	0.600	0.640	0.662	0.654	0.593	0.537	0.605	0.655
14	Henan	0.902	0.875	0.916	0.962	1.017	1.006	1.032	1.022	0.900	1.023	0.966
15	Hubei	0.831	0.850	0.813	0.824	0.900	1.021	1.003	1.012	0.666	0.660	0.858
16	Hunan	0.718	0.749	0.681	0.672	0.763	0.844	1.031	1.064	0.742	0.661	0.792
17	Guangdong	0.671	0.715	0.690	0.664	0.653	0.619	0.617	0.628	0.523	0.567	0.635
18	Guangxi	0.895	1.109	0.897	0.832	0.792	0.741	0.689	0.732	0.660	0.663	0.801
19	Hainan	0.883	1.007	0.909	0.809	0.735	0.686	0.550	0.466	0.178	0.161	0.638
20	Chongqing	1.022	1.011	0.923	0.881	0.891	1.009	0.940	1.032	0.885	0.917	0.951
21	Sichuan	1.052	0.955	0.896	0.872	0.918	1.047	0.950	1.046	0.801	0.786	0.932
22	Guizhou	1.101	1.005	0.762	0.614	0.552	0.582	0.632	0.648	0.512	0.550	0.696
23	Yunnan	1.016	1.029	1.011	1.011	1.015	1.015	0.982	1.013	0.842	0.872	0.981
24	Xizang	1.217	1.041	0.685	1.008	0.618	0.519	0.392	0.221	0.126	0.072	0.590
25	Shaanxi	0.423	0.407	0.401	0.420	0.392	0.432	0.426	0.379	0.240	0.232	0.375
26	Gansu	0.547	0.521	0.452	0.451	0.483	0.502	0.475	0.438	0.377	0.333	0.458
27	Qinghai	1.056	0.927	1.019	0.738	0.824	0.716	0.602	0.550	0.459	0.397	0.729
28	Ningxia	1.046	1.007	1.021	1.006	1.009	1.007	0.692	0.615	0.418	0.387	0.821
29	Xinjiang	0.749	0.865	0.927	1.008	1.005	0.949	0.765	0.822	0.544	0.344	0.798
Mean		0.804	0.778	0.723	0.713	0.708	0.718	0.669	0.655	0.503	0.488	0.676

Bold symbols represent the efficiency value greater than 1.

The calculation of the dependent variable (efficiency of THC health resource allocation) is based on the researches of scholars like Chen, Xu, Yu, etc. (17, 18, 33, 34), which includes five input variables: number of THCs, number of beds in THCs, number of practicing physicians, registered nurses, and pharmacists in THCs; Four output variables: number of diagnosed and treated patients, inpatients, discharged patients in THCs, and bed utilization rate in THCs.

The selection of influencing factors of THC health resource allocation efficiency is based on the researches of Yi, Meng, Tao, and others (35–37). These factors include 13 variables such as the efficiency of total health resource allocation, population density, and the proportion of urban population (represented by X1-X13 in the following text). The calculation methods for the efficiency of total health resource allocation and THC health resource allocation are the same, but their input and output variables differ. The input variables for the efficiency of total health resource allocation include the number of medical and health institutions, the number of health technicians, and the number of hospital beds; while the output variables include the number of diagnosed and treated patients, and the number of discharged patients. Furthermore, the calculation equation for X13 is available in Table 2. The calculation data for each single indicator and the calculation indicator of the composite variables is sourced from the latest 2013–2022 China Health Statistical Yearbook, China Health and Family Planning Statistical Yearbook, and China Statistical Yearbook. Detailed information is presented in Table 2.

**Table 2:** Statistical description of variables included in the analysis of this study.

Category	Variables	Mean	Std. dev.	Minimum	Maximum
Inputs	Number of THCs (units)	1256.74	780.51	133.00	4606.00
	Number of beds in THCs (units)	43540.16	32816.33	2573.00	135705.00
	Number of practicing physicians in THCs (persons)	16136.09	11703.86	559.00	45053.00
	Number of registered nurses in THCs (persons)	11525.91	8965.37	160.00	33728.00
	Number of pharmacists in THCs (persons)	2629.65	1985.91	61.00	7919.00
Outputs	Number of diagnosed and treated patients in THCs (person-times)	37200000.00	30600000.00	2522889.00	126000000.00
	Number of inpatients in THCs (person-times)	1296595.00	1180334.00	8372.00	4895237.00
	Number of discharged patients in THCs (person-times)	1292245.00	1175469.00	8297.00	4875351.00
	Bed utilization rate in THCs (%)	51.32	15.59	9.60	81.10
Dependent variable	(Y) efficiency of THC health resource allocation	0.68	0.27	0.07	1.22
Independent variables (Influencing factors)	(X1) Efficiency of total health resource allocation	0.67	0.19	0.25	1.20
	(X2) Population density (persons/km^2^)	309.44	264.59	2.56	1209.15
	(X3) The proportion of urban population (%)	74.51	292.90	22.87	5042.00
	(X4) Dependency ratio (%)	39.30	6.83	24.98	57.79
	(X5) The proportion of illiterate population (%)	5.99	6.32	1.01	41.18
	(X6) PGDP (CNY)	54919.96	22568.86	5896.00	137039.00
	(X7) Registered urban unemployment rate (%)	3.24	0.56	1.80	4.60
	(X8) Healthcare consumer price index (last year = 100)	102.67	2.43	99.30	115.40
	(X9) *Per capita* disposable income (CNY)	23800.33	7804.61	9746.80	57540.50
	(X10) *Per capita* healthcare expenditure ratio (%)	8.20	2.07	2.63	13.57
	(X11) Public budget expenditures for healthcare (CNY)	1057.44	489.26	391.37	3944.54
	(X12) Passenger traffic volume (10,000 persons)	61846.29	58028.25	825.00	574265.90
	(X13) Elderly population coefficient (%)	10.81	2.75	4.98	18.80

The elderly population coefficient is calculated by dividing the population aged 60 and above by the total population, and then multiplying by 100%.

### 2.2 Super-efficiency SBM model

Data Envelopment Analysis (DEA) serves as a widely used method for assessing efficiency, particularly in evaluating the efficiency or relative efficiency of multiple Decision-Making Units (DMUs) across various organizations such as enterprises, schools, and hospitals (38–40). DEA encompasses several models including CCR models based on constant returns to scale and BCC models based on variable returns to scale, which are commonly referred to as radial DEA models due to their proportional reduction or increase in inputs and outputs. However, the radial distance function is just one of the distance functions, other distance functions such as the farthest distance to the front, the closest distance to the strong efficient front, the closest distance to the weak efficient front, the mixed distance function, and others are also frequently employed in the DEA.

Tone first proposed the SBM model in 2001 (41), which was based on the farthest distance to the front function. This SBM model offers an advantage over traditional radial models like CCR and BCC by integrating the relaxation variable problem into inefficiency measurement (39). Furthermore, in the analysis of DEA models, it is common to have multiple DMUs identified as efficient. This poses a challenge as traditional DEA models yield maximum efficiency values of 1 for all efficient DMUs, making it difficult to distinguish further between efficient efficiencies. To solve this problem, Tone proposed the super-efficiency SBM model in 2002 (42), which has been widely used in various efficiency studies by scholars. The efficiency model for health resource allocation in this study is constructed based on the super-efficiency SBM model with variable returns to scale:


(1) $$\begin{array}{l} \rho =\min\frac{\frac{1}{m}\sum_{i=1}^{m}\bar{x_{i}}/x_{i0}}{\frac{1}{s}\sum_{k=1}^{s}\bar{y_{k}}/y_{k0}}\;\\ s.t.\{\begin{array}{c} \bar{x_{i}}\geq \overset{n}{\underset{j=1,j\neq 0}{\sum }}\lambda_{j}x_{j},\forall i\\ \bar{y_{k}}\leq \overset{n}{\underset{j=1,j\neq 0}{\sum }}\lambda_{j}y_{j},\forall k\\ \bar{x_{i}}\geq x_{i0},y_{k0}\geq \bar{y_{k}}\geq 0,\lambda_{j}\geq 0,\overset{n}{\underset{j=1,j\neq 0}{\sum }}\lambda_{j}=1,\forall i,j,k \end{array} \end{array}$$

This research involves the assessments of health resource allocation efficiency across 29 $\mathrm{DMUs}\left(x_{0},y_{0}\right)$, with each province serving as a DMU. As shown in Eq. (1), ρ signifies the efficiency of the DMU, where the efficiency greater than or equal to 1 indicates efficient, and the efficiency less than 1 denotes inefficient. Each DMU has i types of inputs $x_{i}\left(i=1,2,..,m\right)$, k types of outputs $y_{k}\left(k=1,2,\ldots ,s\right)$, $\bar{x_{i}}$ and $\bar{y_{k}}$ represent the average of all inputs and outputs, respectively. n represents the total number of DMUs, j represents the j-th DMU, m and s represent the total number of input and output variables, i represents the i-th input, k represents the k-th output, λ is the weight, and $\overset{n}{\underset{j=1,j\neq 0}{\sum }}\lambda_{j}=1$ denotes variable returns to scale.

### 2.3 Global Malmquist productivity index

The Malmquist productivity index can dynamically analyze panel data from a time series perspective, which was first established and applied by Caves et al. to measure the level of productivity change (36), and can effectively integrate with DEA theory. The global Malmquist model is a Malmquist index (MI) calculation method proposed by Paster and Lovell (43). It uses the sum of each period as the reference set and has the advantage of cross-period comparability. The common reference set for each period is:


(2) $$\left\{\left(x_{j}^{1},y_{j}^{1}\right)\right\}\cup \left\{\left(x_{j}^{2},y_{j}^{2}\right)\right\}\cup \ldots \cup \left\{\left(x_{j}^{p},y_{j}^{p}\right)\right\}=S^{1}\cup S^{2}\cup \ldots \cup S^{p}=S^{g}$$

In Eq. (2), j represents the j-th DMU; $S^{t}$ is the simplified form of $\left(x_{j}^{t},y_{j}^{t}\right),t=1,2,\ldots ,p$, and since there are 10 periods from 2012 to 2021 in this study, *p* = 10; $S^{g}$ is a common reference set for each period. Therefore, in addition to the utilization of the supper-efficiency SBM model, this study further constructed the global Malmquist productivity index model:


(3) $$M_{g}\left(x^{t+1},y^{t+1},x^{t},y^{t}\right)=\frac{E^{g}\left(x^{t+1},y^{t+1}\right)}{E^{g}\left(x^{t},y^{t}\right)}$$

In Eq. (3), $M_{g}\left(x^{t+1},y^{t+1},x^{t},y^{t}\right)$ is the global Malmquist productivity index, if the index is greater than 1, it indicates an increase in productivity; otherwise, it indicates a decrease in productivity. $E^{g}$ represents the efficiency value of the DMU; $\left(x^{t},y^{t}\right)$ and $\left(x^{t+1},y^{t+1}\right)$ represent the input and output vectors of the DMU during the period_t and period_t + 1, respectively. In addition, MI can also be decomposed into technical efficiency change (EC, representing the relative relationship between actual production and production frontier) and technical change (TC, representing the change in production frontier boundary, that is the situation of technological progress), to comprehensively reflect the productivity change of a DMU from period_t to period_t + 1. The specific formula is shown in Eq. (4):


(4) $$\begin{array}{l} M_{g}\left(x^{t+1},y^{t+1},x^{t},y^{t}\right)=\frac{E^{g}\left(x^{t+1},y^{t+1}\right)}{E^{g}\left(x^{t},y^{t}\right)}\\ \;=\frac{E^{t+1}\left(x^{t+1},y^{t+1}\right)}{E^{t}\left(x^{t},y^{t}\right)}\left(\frac{E^{g}\left(x^{t+1},y^{t+1}\right)}{E^{t+1}\left(x^{t+1},y^{t+1}\right)}\cdot \frac{E^{t}\left(x^{t},y^{t}\right)}{E^{g}\left(x^{t},y^{t}\right)}\right)=EC\times {TC}_{g} \end{array}$$

### 2.4 Spatial auto-correlation

The spatial weight matrix illustrates the level of interdependence among geographical or economic attribute values of spatial section units, serving as a crucial connection between the spatial econometric model in theoretical analysis and the actual spatial effects in the real world (44). Therefore, the choice of spatial econometric model must be based on the suitable spatial weight matrix. Due to the limitations of the national health plan in allocating medical and health resources, and the common use of adjacency spatial weight matrices in spatial studies to demonstrate the mutual influence between neighboring provinces, this article utilizes the Rook geographical adjacency spatial weight matrix as the weight matrix. To prevent missing values in the spatial weight matrix, Hainan is considered adjacent to Guangdong Province due to its geographical proximity and close connection. The specific form is as follows:


(5) $$w_{ij}=\{\begin{array}{c} 1\;\mathrm{when}\;i\;\mathrm{is}\;\mathrm{adjacent}\;to\;j\;\\ 0\;\mathrm{when}\;i\;\mathrm{is}\;\mathrm{not}\;\mathrm{adjacent}\;to\;j\;\mathrm{or}\;i=j \end{array}$$

In Eq. (5), $w_{ij}$ represents the spatial weight matrix between province_i and province_j. There are two forms of spatial auto-correlation: global and local. Global spatial auto-correlation can test the overall spatial distribution pattern of THC health resource allocation efficiency in 29 provinces of China and reflect spatial dependence; local spatial auto-correlation can specifically reflect the degree of spatial correlation between efficiency values in each province and neighboring regions, and identify the contribution of efficiency observations in each spatial unit. The most commonly used global spatial auto-correlation test is the Moran’s Index:


(6) $${\mathrm{Moran}}^{\prime }s\;\mathrm{Index}=\frac{\sum_{i=1}^{n}\sum_{j=1}^{n}w_{ij}\left(x_{i}-\bar{x}\right)\left(y_{i}-\bar{y}\right)}{S^{2}\sum_{i=1}^{n}\sum_{j=1}^{n}w_{ij}}$$

In Eq. (6), $S^{2}=\frac{\sum_{i=1}^{n}\left(x_{i}-\bar{x}\right)}{n}$ represents sample variance; n represents the number of provinces, $x_{i}$, $y_{i}$ represents the efficiency of health resource allocation in province-level THCs, $\bar{x}$, $\bar{y}$ represent the average efficiency; $w_{ij}$ represents $\left(i,j\right)$ element in the spatial weight matrix, and $\overset{n}{\underset{i=1}{\sum }}\overset{n}{\underset{j=1}{\sum }}w_{ij}$ represents the combination of all spatial weight matrices. The value range of Moran’s Index is generally between −1 and 1. When Moran’s Index >0, it indicates a positive spatial auto-correlation. The larger the value, the more pronounced the spatial auto-correlation. When Moran’s Index <0, the spatial auto-correlation is negative. The smaller the value, the greater the spatial difference. When Moran’s Index = 0, the space presents randomness. Furthermore, the Moran scatter diagram is used to reflect the characteristics of local spatial agglomeration, the four quadrants of the Moran scatter diagram are used to identify the relationship between a region and its neighbors. The first and third quadrants represent positive spatial auto-correlation, indicating a similar value agglomeration feature; the second and fourth quadrants represent negative spatial auto-correlation, indicating an opposite value agglomeration feature.

### 2.5 Spatial econometric model

The spatial econometric model addresses the deficiency of traditional econometric models in accounting for spatial correlation arising from geographical distance. It not only assesses the direct impact of influencing factors within a particular region but also evaluates the spillover effect on adjacent regions, that is, the influences of dependent variable and independent variables from other regions on the local dependent variable. The model is expressed as follows:


(7) $$Y_{\mathrm{it}}=\rho w_{i}Y_{t}+\beta X_{\mathrm{it}}+\theta D_{i}X_{t}+\mu_{i}+\gamma_{t}+\varepsilon_{\mathrm{it}},\varepsilon_{\mathrm{it}}=\lambda w_{i}\varepsilon_{t}+\nu_{\mathrm{it}}$$

In Eq. (7), $Y_{\mathrm{it}}$ represents the efficiency of health resource allocation in province-level THCs, $X_{\mathrm{it}}$ is an N*N order explanatory (exogenous) variable matrix, and $w_{i}$ is the i-th row of the N*N order spatial weight matrix; $\theta D_{i}X_{t}$ represents the spatial Weight lag term of the independent variable, ρ is the spatial auto-regressive coefficient, β is the regression coefficient of the independent variable, θ is the spatial regression coefficient of the independent variable, λ is the spatial error regression coefficient, $\mu_{i}$ represents individual effect, $\gamma_{t}$ is the time effect, $\nu_{\mathrm{it}}$ is a random interference term that follows independent identical distribution, i represents province, and t represents year.

**Figure 1:**
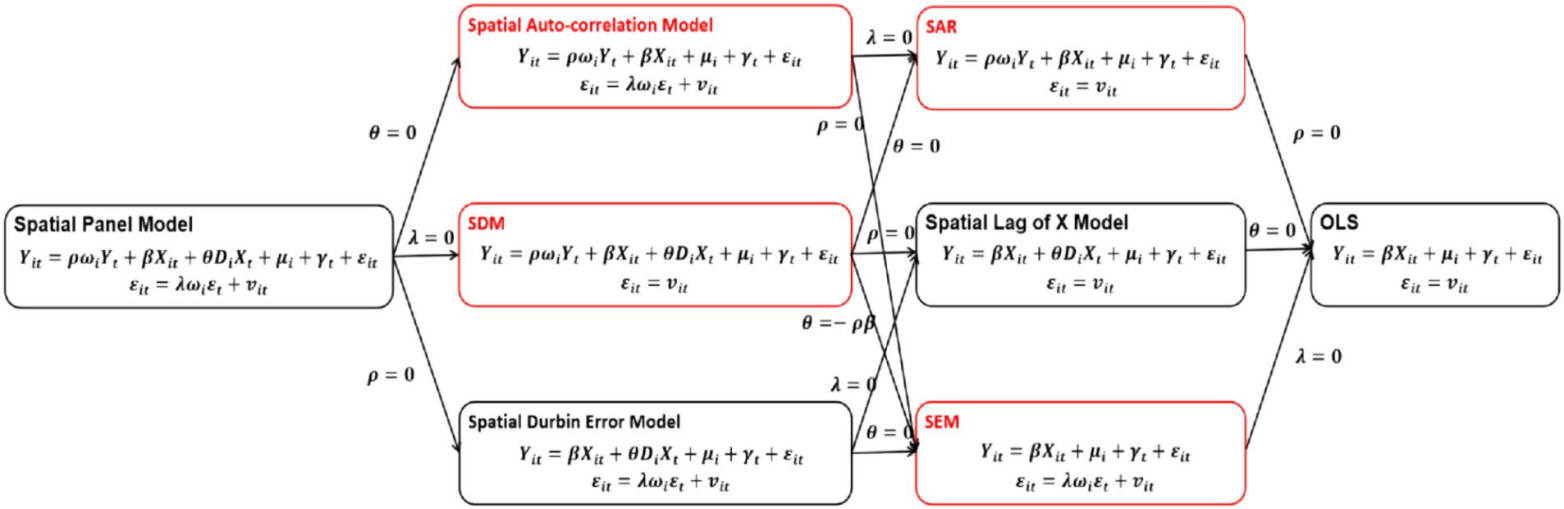
Model selection criteria flowchart. This figure is based on the book *“Advanced Econometrics and Stata Applications”* by Chinese scholar Chen Qiang. The models indicated by the red border in the figure is the spatial econometric models mentioned in the main text.

If λ=0 and θ=0, the spatial econometric model becomes the Spatial Auto-Regression model (SAR), which demonstrates the influence of THC health resource allocation efficiency in other provinces and independent variables on the efficiency of local regions; If ρ=0 and θ=0, it transforms into the Spatial Error Model (SEM), which shows the influence of spatial interference variables and independent variables on the local THC health resource allocation efficiency, excluding spatial independent variables; If θ=0, it represents the spatial auto-correlation model, which encompasses the spatial lag model and the spatial error model; If λ=0, it becomes the Spatial Dubin Model (SDM), simultaneously considering the direct influence of dependent variables and the spatial lag influence of them, both with the influence of THC health resource allocation efficiency in other provinces on the efficiency of local region. In order to better demonstrate the selection criteria for the model, this study created a flowchart based on the research of scholars such as Chen, as shown in Figure 1.

## 3 Results

### 3.1 Efficiency calculation results

This study calculated the THCs’ health resource allocation efficiency values of 29 provinces (autonomous regions, municipalities) in China from 2012 to 2021 by using Matlab R2021b software and a super-efficiency SBM model based on variable returns to scale. The specific results are shown in Table 1.

**Figure 2:**
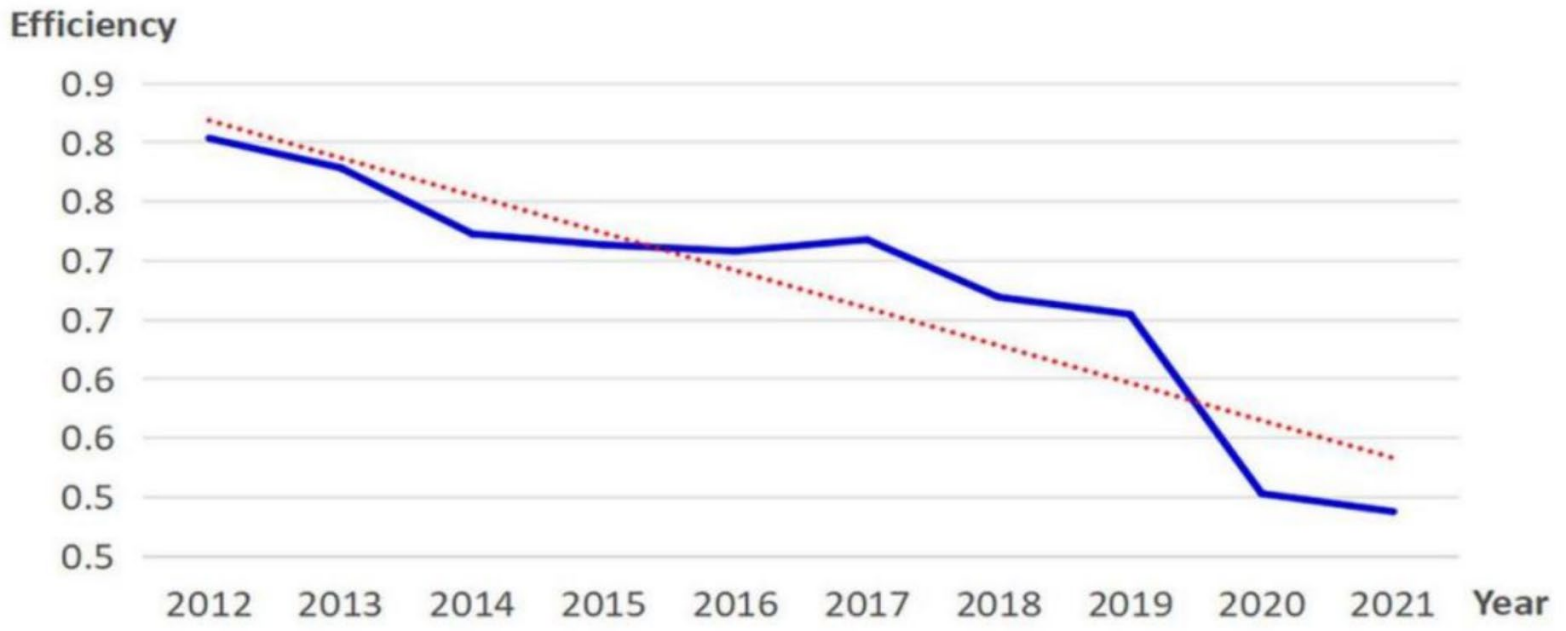
Changes in efficiency of THC health resource allocation nationwide.

The research findings indicate that the mean efficiency of THC health resource allocation in 29 Chinese provinces from 2012 to 2021 is 0.676, which shows the efficiency of THC health resource allocation in various regions generally exhibits DEA inefficiency with overall low efficiency. From a dynamic perspective, the overall THCs’ health resource allocation efficiency in China has displayed a declining trend over the past decade (Figure 2), with the average efficiency dropping from 0.804 in 2012 to 0.488 in 2021. Among them, the mean efficiency reduction between 2019 and 2020 was the largest, which this study speculated may be related to COVID-19.

Specifically, 19 provinces in China, such as Tianjin (8 years), Yunnan (7 years), Zhejiang (6 years), Henan (5 years), Chongqing (4 years), Hubei (3 years), Jiangsu (2 years), Hebei (1 year), etc., have been at the technological forefront in terms of THCs’ health resource allocation efficiency in some years. The remaining 10 provinces have not been at the technological forefront once during this decade. Moreover, there is a notable disparity in the efficiency of THC health resource allocation among different regions in China: only Tianjin Province has an average efficiency greater than 1, 9 regions such as Yunnan and Henan have an average efficiency between 0.8 and 1, and 10 regions such as Xinjiang and Hunan have an average efficiency between 0.6 and 0.8. The average efficiency values of the other 9 provinces are all below 0.6, indicating that there is a notable disparity in the average efficiency values among different provinces. This fact is also reflected in the comparison of the top and bottom three regions in terms of average efficiency. The top three regions are Tianjin (1.002), Yunnan (0.981), and Henan (0.966), while the bottom three regions are Jilin (0.208), Inner Mongolia (0.281), and Shanxi (0.287), demonstrating a significant difference in average efficiency values.

### 3.2 Productivity changes

**Figure 3:**
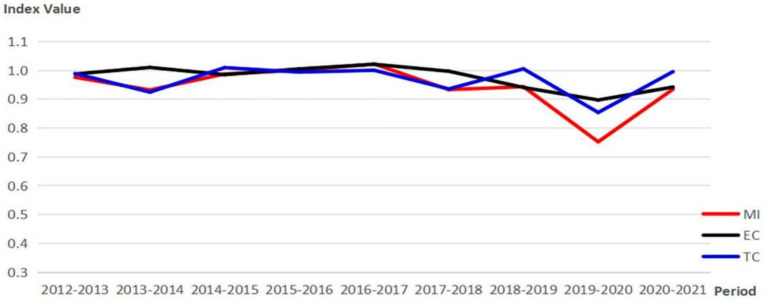
Changes and decomposition of the Malmquist Index.

This study also combined the super-efficiency SBM model with the global Malmquist model to calculate the Malmquist index (MI) of THCs’ health resource allocation efficiency and its decomposition indicators in 29 provincial administrative regions of China from 2012 to 2021. Then, dynamic analysis and evaluation were conducted separately. The specific results are shown in Figure 3.

According to Figure 3, the Malmquist Index (MI) of THCs’ health resource allocation efficiency in China has remained around 1 from 2012 to 2021, indicating relatively stable global Malmquist productivity of THCs in China over the past decade. During the period of sudden decline in MI from 2019 to 2020, MI was 75.2%, EC was 89.7%, and TC was 85.4%, suggesting that the decrease in global Malmquist productivity of THCs in China during the COVID-19 period is likely limited by both technology level and scale efficiency. At the provincial level, the average MI, EC, and TC of THCs’ health resource allocation efficiency in 29 provinces in China are 0.942, 0.976, and 0.968, this indicates that the productivity of THCs in China has been declining over the past decade. Details are illustrated in Table 3.

**Table 3:** MI and its decomposition by province.

DMU	Regions	MI	EC	TC
1	Tianjin	1.000	1.009	0.992
2	Hebei	0.878	0.914	0.991
3	Shanxi	0.901	0.958	0.944
4	Inner Mongolia	0.926	0.985	0.944
5	Liaoning	0.920	0.969	0.950
6	Jilin	0.918	0.972	0.944
7	Heilongjiang	0.916	0.971	0.942
8	Jiangsu	1.015	1.004	1.010
9	Zhejiang	0.971	1.003	0.967
10	Anhui	0.953	0.961	0.994
11	Fujian	0.935	0.968	0.965
12	Jiangxi	0.934	0.962	0.988
13	Shandong	0.956	0.969	1.000
14	Henan	1.016	1.007	1.010
15	Hubei	0.984	0.998	0.984
16	Hunan	1.002	1.002	1.001
17	Guangdong	0.984	0.976	1.013
18	Guangxi	0.973	0.999	0.972
19	Hainan	0.857	0.892	0.964
20	Chongqing	0.992	1.031	0.963
21	Sichuan	0.974	0.991	0.982
22	Guizhou	0.934	0.942	0.995
23	Yunnan	0.985	1.000	0.985
24	Xizang	0.767	0.898	0.854
25	Shaanxi	0.945	0.982	0.962
26	Gansu	0.949	0.988	0.961
27	Qinghai	0.905	1.009	0.894
28	Ningxia	0.905	1.001	0.904
29	Xinjiang	0.937	0.955	0.988
Mean		0.942	0.976	0.968

Bold symbols represent values greater than 1.

### 3.3 Spatial correlation test

#### 3.3.1 Global spatial auto-correlation

Based on Table 4, it is evident that despite the fluctuations in Moran’s index of THCs’ health resource allocation efficiency, it remains consistently positive (with an average of 0.238). Except for 2016 and 2017, Moran’s index has surpassed the 10% significance level test over the past 8 years. These suggest that the spatial distribution of THCs’ health resource allocation efficiency across 29 provinces in China exhibits significant positive spatial auto-correlation characteristics over the last decade, with relatively stable spatial dependence.

**Table 4:** Global Moran index.

Year	Moran’s index	Z	*p*-value
2012	0.360^***^	3.202	0.001
2013	0.376^***^	3.345	0.001
2014	0.238^**^	2.217	0.027
2015	0.258^**^	2.377	0.018
2016	0.135	1.379	0.168
2017	0.126	1.298	0.194
2018	0.258^**^	2.363	0.018
2019	0.241^**^	2.220	0.026
2020	0.203^*^	1.930	0.054
2021	0.186^*^	1.789	0.074

^*^p-value < 0.1, ^**^p-value < 0.05, ^***^p-value < 0.01.

#### 3.3.2 Local spatial auto-correlation

**Figure 4:**
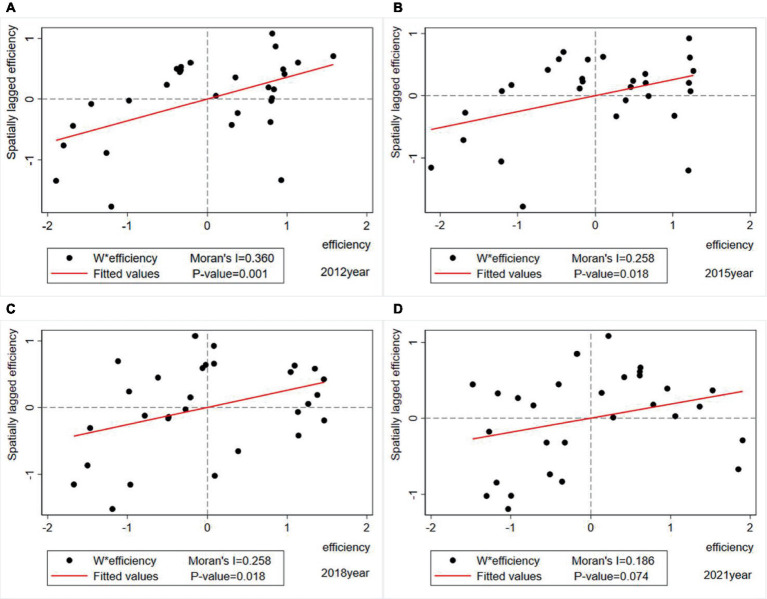
Moran scatter diagram of THCs’ health resource allocation efficiency. **(A)** 2012 Moran scatter diagram. **(B)** 2015 Moran scatter diagram. **(C)** 2018 Moran scatter diagram. **(D)** 2021 Moran scatter diagram.

To examine the spatial correlation of a specific region, this study presents a Moran scatter diagram of THCs’ health resource allocation efficiency. As shown in Figure 4, only 2012, 2015, 2018, and 2021 are selected as representative years, where each scatter represents a DMU.

The Moran scatter diagram categorizes the THCs’ health resource allocation efficiency clusters of 29 provinces into four quadrants: The first quadrant (High-High) indicates that the high-efficiency province is surrounded by other regions that are also high-efficiency; The second quadrant (Low-High) indicates that the low-efficiency province is surrounded by other regions with high-efficiency; The third quadrant (Low-Low) indicates that the low-efficiency province is surrounded by other regions that are also low-efficiency; The fourth quadrant (High-Low) indicates that the high-efficiency province is surrounded by other regions with low-efficiency. The first and third quadrants show positive spatial auto-correlation, while the second and fourth quadrants show negative spatial auto-correlation. The scatter diagram results show that most regions were located in the first quadrant (High-High) and third quadrant (Low-Low), indicating a significant positive spatial auto-correlation in the efficiency of THC health resource allocation, which is consistent with the global test results. Overall, the Moran scatter points of THCs’ health resource allocation efficiency in various provinces were 55.17, 55.17, 51.72, and 65.52% of the total sample in 2012, 2015, 2018, and 2021 year, respectively (see Table 5).

**Table 5:** Cluster test for the efficiency of THC health resource allocation.

Year	Spatial aggregation		Spatial discretization	
	High-High (I)	Low-Low (III)	Low-High (II)	High-Low (IV)
2012	Tianjin, Shandong, Guangxi, Chongqing, Sichuan, Guizhou, Yunnan, Xizang, Qinghai (9)	Shanxi, Inner Mongolia, Liaoning, Jilin, Heilongjiang, Shaanxi, Gansu (7)	Jiangsu, Anhui, Fujian, Hunan, Guangdong, Xinjiang (6)	Hebei, Zhejiang, Jiangxi, Henan, Hubei, Hainan, Ningxia (7)
2015	Tianjin, Jiangsu, Hubei, Guangxi, Sichuan, Yunnan, Xizang, Qinghai, Xinjiang (9)	Shanxi, Inner Mongolia, Liaoning, Jilin, Heilongjiang, Guangdong, Shaanxi (7)	Anhui, Fujian, Jiangxi, Shandong, Hunan, Guizhou, Gansu (7)	Hebei, Zhejiang, Henan, Hainan, Chongqing, Ningxia (6)
2018	Jiangsu, Zhejiang, Anhui, Hubei, Hunan, Guangxi, Chongqing (7)	Hebei, Shanxi, Inner Mongolia, Liaoning, Jilin, Heilongjiang, Gansu, Qinghai (8)	Fujian, Jiangxi, Shandong, Guangdong, Guizhou, Xizang, Shaanxi (7)	Tianjin, Henan, Hainan, Sichuan, Yunnan, Ningxia, Xinjiang (7)
2021	Jiangsu, Zhejiang, Jiangxi, Shandong, Hubei, Hunan, Guangxi, Chongqing, Guizhou, Yunnan (10)	Shanxi, Inner Mongolia, Liaoning, Jilin, Heilongjiang, Gansu, Qinghai, Ningxia, Xinjiang (9)	Hebei, Anhui, Fujian, Hainan, Xizang, Shaanxi (6)	Tianjin, Henan, Guangdong, Sichuan (4)

Arabic numerals in parentheses represent the number of provinces, cities, and districts.

### 3.4 Optimal model selection

#### 3.4.1 LM test

This study first made a preliminary judgment on the model selection through the widely used LM test in spatial econometric research. Both robust tests of Spatial Error and Spatial lag, displayed in Table 6, rejected the null hypothesis at a significance level of 1%, indicating that the selected sample has a dual effect of spatial lag and spatial error. Therefore, it is determined that SEM and SAR models are meaningful in this study and all SDM, SEM, and SAR models can be utilized in this study.

**Table 6:** LM test.

Test		Statistic	Degree of freedom	*p*-value
Spatial error	Lagrange multiplier	1.675	1.00	0.196
	Robust Lagrange multiplier	7.409	1.00	0.006
Spatial lag	Lagrange multiplier	5.301	1.00	0.021
	Robust Lagrange multiplier	11.035	1.00	0.001

#### 3.4.2 Degradation test

As shown in Table 7, this study conducted a degradation test on the SDM model through the combination of LR and Wald tests, and the results showed that the SDM model did not degrade into either SAR or SEM models. In addition, the Hausman test results indicated that a fixed effects model should be used; the LR test results also showed that a time and individual dual fixed effects model should be selected. Consequently, so this study selected the SDM model with time and individual dual fixed effects as the optimal spatial econometric model for this study.

**Table 7:** LR, Wald, and Hausman tests.

Test		χ2	Degree of freedom	*p*-value
LR test	Individual fixed	37.180	24	0.042
	Time fixed	304.790	24	0.000
	Degradation test for SAR	58.140	13	0.000
	Degradation test for SEM	55.730	13	0.000
Wald test	Degradation test for SAR	63.87	11	0.000
	Degradation test for SEM	61.94	13	0.000
Hausman test		45.190	10	0.000

### 3.5 Regression results and spatial effect decomposition

This research utilized Stata software and three spatial econometric models, SDM, SEM, and SAR, to perform spatial regression on the THCs’ health resource allocation efficiency across 29 provinces in China. The findings are presented in Table 8. The regression results revealed that the SDM model exhibited the lowest σ^2 and the highest goodness of fit R^2, making it the optimal model with the highest goodness of fit among the three models. The spatial auto-regression coefficients of three models were all negative and significant, indicating a substantial spatial negative auto-correlation in the efficiency of THC health resource allocation in China. This implies that the change in the efficiency of THC health resource allocation in a province will cause a change in the efficiency of THC health resource allocation in its neighboring provinces, and this change generally shows an inhibitory effect. Moreover, the regression results of the optimal model SDM indicated that factors such as the efficiency of total health resource allocation, population density, PGDP, urban unemployment rate, *per capita* disposable income, *per capita* healthcare expenditure ratio, public budget expenditures for healthcare, and passenger traffic volume all have varying degrees of impact on the efficiency of THC health resource allocation.

**Table 8:** Regression results of spatial econometric models.

Variable	SDM		SAR		SEM	
	Coefficient	*p*-value	Coefficient	*p*-value	Coefficient	*P*-value
X1	0.439881 3^***^	0.000	0.318698 2^***^	0.000	0.314158 3^***^	0.000
X2	−0.001976 7^**^	0.012	−0.002147 5^***^	0.006	−0.001984 3^**^	0.011
X3	0.000018 4	0.285	0.000029 2	0.111	0.000025 8	0.157
X4	0.004530 4	0.221	0.007096 1^*^	0.071	0.006546 0^*^	0.086
X5	−0.003603 2	0.533	−0.010137 1^*^	0.092	−0.008927 2	0.125
X6	0.000002 6^***^	0.005	0.000002 8^***^	0.003	0.000003 2^***^	0.001
X7	−0.043971 7^***^	0.007	−0.021269 8	0.186	−0.033289 2^**^	0.048
X8	0.001025 1	0.722	−0.000735 6	0.806	−0.001072 6	0.712
X9	0.000017 9^***^	0.001	0.000011 6^**^	0.010	0.000007 3	0.124
X10	0.009840 7	0.279	0.017688 8^*^	0.053	0.021400 9^**^	0.022
X11	−0.000330 5^***^	0.000	−0.000321 8^***^	0.000	−0.000308 4^***^	0.000
X12	−0.000000 3^*^	0.071	−0.000000 4^**^	0.033	−0.000000 4^**^	0.044
X13	0.015681 3	0.151	0.000253 4	0.981	0.001004 4	0.922
W*X1	−0.026378 2	0.854	–	–	–	–
W*X2	0.004133 2^**^	0.015	–	–	–	–
W*X3	−0.000049 8	0.279	–	–	–	–
W*X4	0.001228 0	0.873	–	–	–	–
W*X5	0.008916 8	0.488	–	–	–	–
W*X6	0.000002 5	0.199	–	–	–	–
W*X7	−0.226241 0^***^	0.000	–	–	–	–
W*X8	0.005591 5	0.367	–	–	–	–
W*X9	−0.000048 9^***^	0.000	–	–	–	–
W*X10	0.055752 5^***^	0.007	–	–	–	–
W*X11	0.000041 0	0.639	–	–	–	–
W*X12	0.000000 1	0.768	–	–	–	–
W*X13	0.025130 9	0.248	–	–	–	–
ρ/λ	−0.155540 3^*^	0.084	−0.154292 9^**^	0.045	−0.252842 0^**^	0.010
σ^2	0.005787 5^***^	0.000	0.007064 0^***^	0.000	0.006945 8^***^	0.000
R^2	0.414		0.162		0.213	

^*^p-value < 0.1, ^**^p-value < 0.05, ^***^p-value < 0.01.

Although SDM explains the economic spatial correlation between provinces, the estimation results cannot directly reflect the direct effects and indirect effects due to the inclusion of spatial lag effects between dependent and independent variables in SDM. Therefore, it is necessary to calculate the partial calculus of SDM and decompose the direct and indirect effects of independent variables (45). The direct effect describes the direct impact of the independent variables on the efficiency in local or neighboring provinces; The indirect effect shows the indirect impact of the independent variables on efficiency within a province through neighboring provinces. The specific effect decomposition results are shown in Table 9.

**Table 9:** Spatial effect decomposition.

Variable	LR_Direct	*P* > z	LR_Indirect	*P* > z	LR_Total	*P* > z
X1	0.443793 1^***^	0.000	−0.068515 4	0.613	0.375277 7^***^	0.005
X2	−0.002201 8^***^	0.001	0.003971 1^**^	0.012	0.001769 3	0.280
X3	0.000021 3	0.248	−0.000044 4	0.275	−0.000023 1	0.573
X4	0.004738 6	0.248	0.001109 8	0.863	0.005848 4	0.409
X5	−0.005078 2	0.460	0.009615 9	0.387	0.004537 7	0.704
X6	0.000002 5^***^	0.004	0.000001 6	0.327	0.000004 2^**^	0.014
X7	−0.036314 6^**^	0.023	−0.195882 4^***^	0.000	−0.232197 1^***^	0.000
X8	0.000454 5	0.870	0.004319 1	0.446	0.004773 6	0.354
X9	0.000019 6^***^	0.000	−0.000046 2^***^	0.000	−0.000026 7^***^	0.006
X10	0.007879 1	0.414	0.047212 8^***^	0.006	0.055091 9^***^	0.008
X11	−0.000334 4^***^	0.000	0.000081 5	0.297	−0.000252 9^***^	0.003
X12	−0.000000 3^*^	0.081	0.000000 1	0.639	−0.000000 2	0.393
X13	0.014028 4	0.183	0.017311 4	0.360	0.031339 8	0.109

^*^p-value < 0.1, ^**^p-value < 0.05, ^***^p-value < 0.01.

## 4 Discussion

### 4.1 Coexistence of inefficiencies and differences

The research findings showed a continuous decline in the average efficiency of THC health resource allocation in China from 2012 to 2021, dropping from 0.804 in 2012 to 0.488 in 2021. This study suggests that this decline may be attributed to three factors. Firstly, it may be associated with the disorderly expansion of urban large hospitals in China over the past decade. According to the data displayed in the China Health Statistical Yearbook, the growth rate of the total number of beds in overall medical institutions is much higher than that of township health centers (46). Furthermore, scholarly evidence suggests that the expansion of urban large hospitals has resulted in an influx of rural patients (47), thereby reducing the proportion of patients in township health centers and diminishing rural efficiency.

Secondly, this may be related to the overall increase in the urbanization rate. With the unrelenting improvement of the urbanization rate in China (48, 49), the development of various conditions in urban areas, including medical and healthcare, surpass those in rural areas. In addition, the current living conditions of people are getting better and the *per capita* disposable income is constantly increasing (50). As a result, more and more residents are inclined to seek medical treatment at large urban hospitals with more advanced medical and health technologies when they are sick, resulting in the shrinkage of THCs’ healthcare services.

Thirdly, such results may indeed demonstrate the fact that there is room for improvement in the efficiency of THC health resource allocation in rural areas of China. That is, the current hierarchical diagnosis and treatment system in China is not perfect, and an orderly medical pattern has not yet been formed. Nowadays, many large tertiary hospitals in cities have utilized medical and health resources that should have been used to diagnose and treat common diseases in grassroots medical institutions, resulting in unclear division of labor and low efficiency among institutions. Many studies have also indicated that there is indeed a problem of low efficiency in the allocation of rural health resources in China. For example, scholars such as Feng and Liu have concluded in their studies that the overall efficiency level of rural public health resource allocation in China is relatively low (31, 51). Chen and others have also written a research paper specifically on how to improve the uneven distribution of medical resources between urban and rural areas in China (52).

The research results also show that over one-third of the provinces in China have never reached the technological forefront in terms of THCs’ health resource allocation efficiency during the past decade, and there are also 9 provinces with average efficiencies below 0.6 over the past decade. These indicate that many regions in China’s 29 provinces have been in a relatively inefficient state of THCs’ health resource allocation efficiency for a long time. In addition, this study has also identified significant disparities in the efficiency of THC health resource allocation across different provinces in China. For instance, over the last decade, Tianjin province has displayed the highest efficiency in THCs’ health resource allocation, averaging 1.002, whereas Jilin province has exhibited the lowest efficiency, with an average of only 0.208. The disparity in THCs’ health resource allocation efficiency between these two provinces is nearly fivefold, indicating a significant difference in resource allocation among provinces, which may be related to the local economic and social development levels. What’s more, this study suggests focusing on the input aspects such as the number of beds, physicians, registered nurses, and pharmaceuticals in THCs, and the output aspects such as the number of diagnosed and treated patients, inpatients, discharged patients, and bed utilization rate in THCs can enhance the overall efficiency of THC health resource allocation in China.

### 4.2 The overall decline in productivity

The research findings indicate that over the period from 2012 to 2021, only four provinces in China (Tianjin, Jiangsu, Henan, and Hunan) exhibited average MI values equal to or greater than 1 out of the 29 provinces. The efficiency growth in Jiangsu, Henan, and Hunan provinces is found to be influenced comprehensively by scale efficiency and technological progress, while the primary factor affecting the efficiency growth in Tianjin is the improvement of scale efficiency. The remaining 25 provinces experienced a global Malmquist productivity of less than 1 over these 10 years, suggesting a declining trend in the efficiency of THC health resource allocation in the majority of Chinese provinces during the sample period. Provinces with average MI values below 1, such as Zhejiang, Chongqing, Yunnan, Qinghai, and Ningxia, are primarily impacted by technological decline. Shandong and Guangdong provinces are mainly affected by the decline in scale efficiency. Eighteen provinces, including Hebei, Shanxi, Inner Mongolia, Liaoning, Jilin, Heilongjiang, Anhui, Fujian, Jiangxi, Hubei, Guangxi, Hainan, Sichuan, Guichou, Xizang, Shanxi, Gansu, and Xinjiang, have been affected by both technological decline and a decline in scale efficiency. Furthermore, in terms of MI ranking, Henan (1.016), Jiangsu (1.015), and Hunan (1.002) are the top three, while Xizang (0.767), Hainan (0.857), and Hebei (0.878) are the bottom three. These results not only indicate that the efficiency of THC health resource allocation in some provinces is somewhat constrained but also suggest that there is a certain gap in the productivity of THCs among provinces.

The overall productivity of THCs in various provinces of China is declining, as the average Malmquist index of 29 provinces is 0.942 over the past decade, indicating an average decrease of 3.2% in THCs’ productivity nationwide. Moreover, the decomposition index EC of MI is 0.976 and TC is 0.968, indicating the declining efficiency of THC health resource allocation in China is due to its change in productivity being both “non-scale efficient” and “non-technical efficient,” which results in the problem of insufficient investment scale and inefficiency coexisting in the allocation of THCs’ health resources.

Therefore, this study suggests that THCs can not only improve the efficiency of health resource allocation in rural areas from the perspective of input–output but also enhance it through technological progress and scientific management (53). In terms of technological progress, each THC should focus on enhancing its technological capabilities, which can be achieved through scientific methods such as strengthening its treatment technology advantages, improving disease prevention and control technologies, promoting rehabilitation technological efficacy, and completely improving the utilization efficiency of health resources. In the complex situation of low efficiency coexists with both redundant and insufficient in investment (54), government departments need to carefully consider the efficiency of resource allocation in reality, and allocate high-quality health resources reasonably according to needs. For example, when providing health financial funding to THCs in various provinces, the focus of investment should be adjusted to improve resource utilization efficiency, avoiding blind expansion and redundancy of health resource investment scale (55).

### 4.3 Spatial auto-correlation and agglomeration

From a global spatial auto-correlation perspective, despite fluctuations in Moran’s index of THCs’ health resource allocation efficiency in China between 2012 and 2021, all values remained positive (with a mean of 0.238). Moreover, 80% of the Moran’s index values over the past decade passed the significance test. This implies there is a significant positive spatial auto-correlation in the THCs’ health resource allocation efficiency across the 29 provinces in China, with relatively stable spatial dependence. This spatial interdependence is also evident in the regression analysis of efficiency. The negative spatial auto-regressive coefficients in three selected spatial econometric models (SDM, SAR, and SEM) all rejected the null hypothesis at a 10% significance level. This suggests that the changes in THCs’ health resource allocation efficiency in one province will impact neighboring provinces and exert inhibitory effects overall.

Furthermore, through local spatial auto-correlation tests like the Moran scatter diagram and cluster tests, this research illustrates the specific spatial clustering of THCs’ health resource allocation efficiency in various Chinese provinces more accurately. These findings revealed the spatial agglomerations of THCs’ health resource allocation efficiency in China’s provinces with an increasing positive spatial auto-correlation trend over these years. However, there are specific differences in the spatial aggregation and spatial discretization quadrant of each province. For example, in the quadrant of spatial aggregation, Guangxi Province has remained stable in the first quadrant (High-High) over these 4 years, indicating that the THCs’ health resource allocation efficiency of Guangxi Province is efficient and surrounded by other provinces that are also efficient; Shanxi, Inner Mongolia, Liaoning, Jilin, and Heilongjiang province are stably in the third quadrant (Low-Low), indicating that the THCs’ health resource allocation efficiency in these regions is inefficient and surrounded by other provinces that are also inefficient. In the quadrant of spatial discretization, Fujian Province has remained stable in the second quadrant (Low-High) for these 4 years, indicating that the THCs’ health resource allocation efficiency of Fujian Province is low, but it is surrounded by other provinces with high efficiency; Henan Province remains steady in the fourth quadrant (High-Low), indicating that the THCs’ health resource allocation efficiency of the Henan Province is high, but it is surrounded by other provinces with low efficiency.

To promote the balanced development of rural healthcare in China, strategies like enhancing inter-provincial communication (56) and establishing a holistic health service data management system can be implemented to create a network framework that spans horizontally, vertically, and to the peripheries (57). This approach aims to mitigate resource disparities among regions and foster a collaborative health resource mechanism with neighboring areas through the efficient allocation of health resources (58).

### 4.4 Multivariate influencing factors and spatial spillover effects

The regression findings from the optimal spatial econometric model SDM in this research indicate that there is a significant negative spatial auto-correlation in China’s THCs’ health resource allocation efficiency. The spatial auto-regression coefficient for THCs’ health resource allocation efficiency in China from 2012 to 2021 is *ρ* = −0.1555403, passing the significance test at 10%. This implies that the efficiency of THC health resource allocation in one province can impact the efficiency of surrounding areas. Therefore, provinces aiming to enhance their health resource allocation efficiency should fully consider the spatial spillover effects from neighboring high-efficiency regions to maximize the spatial impact on THCs’ health resource allocation efficiency and enhance the overall national health resource allocation efficiency.

The explanatory mechanism of this study is to select representative indicators from multiple perspectives such as society, economy, culture, and education to study the influencing factors of the efficiency of health resource allocation in THCs. Under this mechanism, we selected 13 influencing factors based on the extensive spatial econometric research literature conducted by scholars such as Yi, Meng, Tao, and others in the field of health resource allocation. From the results of the regression, the coefficients of the efficiency of total health resource allocation and PGDP are positive and both reject the null hypothesis at a significance level of 1%. This means that the higher the efficiency of total health resource allocation and PGDP, the higher the efficiency of THC health resource allocation in the local province. The coefficients of public budget expenditures for healthcare and passenger traffic volume are negative and significant, indicating that these influencing factors will have an inhibitory effect on the improvement of THCs’ health resource allocation efficiency in the local province. The spatial lag coefficient of the *per capita* healthcare expenditure ratio is positive and rejects the null hypothesis at a significance level of 1%, indicating that the higher the healthcare expenditure ratio in neighboring provinces, the higher the efficiency of THC health resource allocation in the local province. The coefficient of population density is negative and significant, while the coefficient of the spatial lag term is significantly positive. This indicates that the improvement of population density in a province will have an inhibitory effect on the efficiency of THC health resource allocation itself, and have a significant promoting effect on the efficiency of THC health resource allocation in neighboring provinces. The coefficient and spatial lag coefficient of the registered urban unemployment rate are both negative and through hypothesis testing at a significance level of 1%, it is shown that the urban unemployment rate has a significant spatial inhibitory effect and effectively controls the unemployment rate will promote the efficiency of THC health resource allocation in both local province and neighboring provinces. The coefficient of *per capita* disposable income is positive while the coefficient of its spatial lag term is negative, and both coefficients reject the null hypothesis at a significance level of 1%. This indicates that the improvement of disposable income in a province will not only promote the efficiency of THC health resource allocation itself but also have a significant inhibitory effect on the improvement of THCs’ health resource allocation efficiency in neighboring provinces.

In addition, this study also decomposed the spatial effects of various influencing factors on the efficiency of THC health resource allocation. From a direct effect perspective, for every 1% increase in the efficiency of total health resource allocation, PGDP, and *per capita* disposable income, the THCs’ health resource allocation efficiency in local province increases by 44.3793, 0.0003, and 0.0020%, respectively. Whenever the population density, urban unemployment rate, public budget expenditures for healthcare, and passenger traffic volume increase by 1%, the THCs’ health resource allocation efficiency in the local province decreases by 0.2202, 3.6315, 0.0334, and 0.00003%, respectively. From the perspective of indirect effects, for every 1% increase in the population density and *per capita* healthcare expenditure ratio in neighboring areas, the efficiency of THC health resource allocation in the local province will increase by 0.3971 and 4.7213%, respectively. Whenever the urban unemployment rate and *per capita* disposable income in neighboring areas increase by 1%, the THCs’ health resource allocation efficiency in the local province will decrease by 19.5882 and 0.0046%, respectively. From the total effect results of the interaction and cancellation between direct and indirect effects, the efficiency of total health resource allocation, PGDP, and *per capita* healthcare expenditure ratio increasing by 1% will, respectively, increase the THCs’ health resource allocation efficiency by 37.5278, 0.0042, and 5.5092%. Whenever the registered urban unemployment rate, *per capita* disposable income, and public budget expenditures for healthcare increase by 1%, the efficiency of THC health resource allocation will decrease by 23.2197, 0.0027, and 0.0253%, respectively. To sum up, the efficiency of total health resource allocation, population density, PGDP, urban unemployment rate, per capital disposable income, per capital health expenditure ratio, public health budget, and passenger traffic volume are the key factors that affect the efficiency of health resource allocation in THCs. Therefore, Chinese provinces should fully consider and play the role of these influencing factors when improving the efficiency of health resource allocation in township health centers.

### 4.5 Insufficiencies

Firstly, there is still room for further improvement in the selection of variables. In theory, the healthcare market has the characteristics of multiple inputs and outputs with both expected and unexpected outputs coexisting (59). However, this study has not included some unexpected outputs such as medical expenses of outpatients and inpatients in the calculation of efficiency values yet, and some indicators that have been considered cannot comprehensively summarize the input–output situation of THCs.

Secondly, China’s complete rural medical and healthcare network is a three-level healthcare structure that includes county-level hospitals, maternal and child health centers, disease prevention and control centers; township-level township health centers; village-level health centers, clinics, individual clinics, and many other institutions. However, this study only selected rural medical and health institutions THCs at the township level as the research objects, so the results of this study can only serve as a typical representative of the medical and health situation in rural areas of China.

Thirdly, this study found that the efficiency of total health resource allocation, which includes both urban and rural areas, has a significant impact on the efficiency of health resource allocation in rural TCH. We believe that the health resource allocation efficiency in urban areas will have an impact on the efficiency of rural health resource allocation. Although a brief discussion on this impact was included in the discussion section of this study, we are still unable to make a specific quantitative analysis, due to the inaccurate data. Future research on this topic may be considered.

## Data availability statement

The datasets presented in this study can be found in online repositories. The names of the repository/repositories and accession number(s) can be found at: http://www.stats.gov.cn/sj/ndsj/.

## Author contributions

NM: Conceptualization, Data curation, Formal analysis, Investigation, Methodology, Resources, Supervision, Validation, Visualization, Writing – original draft, Writing – review & editing. KS: Formal analysis, Validation, Writing – review & editing. XZ: Methodology, Conceptualization, Writing – review & editing. CL: Validation, Conceptualization, Writing – review & editing. XL: Data curation, Writing – review & editing. TP: Conceptualization, Writing – review & editing. DW: Formal analysis, Writing – review & editing. XM: Formal analysis, Methodology, Supervision, Writing – review & editing, Funding acquisition, Conceptualization, Project administration.
